# Nonlinear system identification of receptive fields from spiking neuron data

**DOI:** 10.1186/1471-2202-16-S1-P46

**Published:** 2015-12-18

**Authors:** Dorian Florescu, Daniel Coca

**Affiliations:** 1 Department of Automatic Control and Systems Engineering, University of Sheffield, Sheffield, S1 3JD, UK

## 

Identification of a linear filter in cascade with a spiking neuron has been previously considered [1] under the assumption that the input of the Hodgkin-Huxley spiking neuron can be measured in addition to the input and output of the circuit. An alternative method to identify a linear filter given the input to the circuit and the spike time sequence, has been proposed [2] for Integrate-and-Fire (IAF) neurons. The method was further extended [3] to nonlinear receptive fields that can be described by a Volterra series.

**Figure 1:**
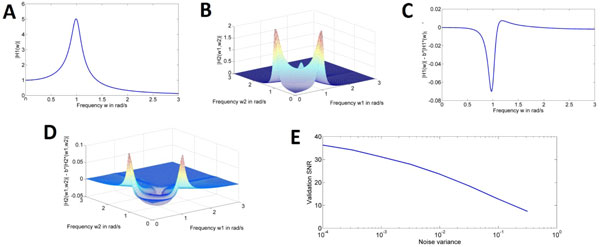
**A&B - GFRF functions of the original filter, C&D - Difference between the original GFRFs and the scaled versions for the identified filter, E - SNR between the validation signal and the identified filter output**.

Here we propose a new system identification methodology to identify a more general NARMAX representation of the nonlinear receptive field in cascade with an ideal-IAF neuron, based only on measurements of the input stimulus and the spike time sequence of the IAF neuron. By using an orthogonal forward selection algorithm we are able to derive the NARMAX representation of a scaled version of the nonlinear filter directly from the reconstructed input of an ideal IAF neuron. The method is further extended to leaky-IAF neurons which require estimating an additional spiking neuron parameter. We use the NARMAX methodology to identify recursively the nonlinear filter and the spiking neuron parameter in the presence of noise. Statistical and dynamical model validation tests are used to check if the identified nonlinear filter models are an adequate representation of the underlying nonlinear information processing mechanisms. The performance and robustness to noise of the proposed methods is demonstrated through numerical simulation studies. Specifically, we compared the generalized (higher-order) frequency response functions (GFRFs) of the original and the identified nonlinear filter (Figures 1 A-D) and evaluated, for different noise levels, the SNR of the model predicted output on a validation data set (Figure 1 E).
